# Supplementary material for: The Flexible, Extended Coil of the PDZ-Binding Motif of the Three Deadly Human Coronavirus E Proteins Plays a Role in Pathogenicity
Source: Viruses. 2022 Aug 2;14(8):1707. doi: 10.3390/v14081707 (PMC9416557; doi:10.3390/v14081707)
Supplement: Supplementary file 1 [file viruses-14-01707-s001.zip › viruses-1792893-supplementary.pdf]

Supplementary Materials

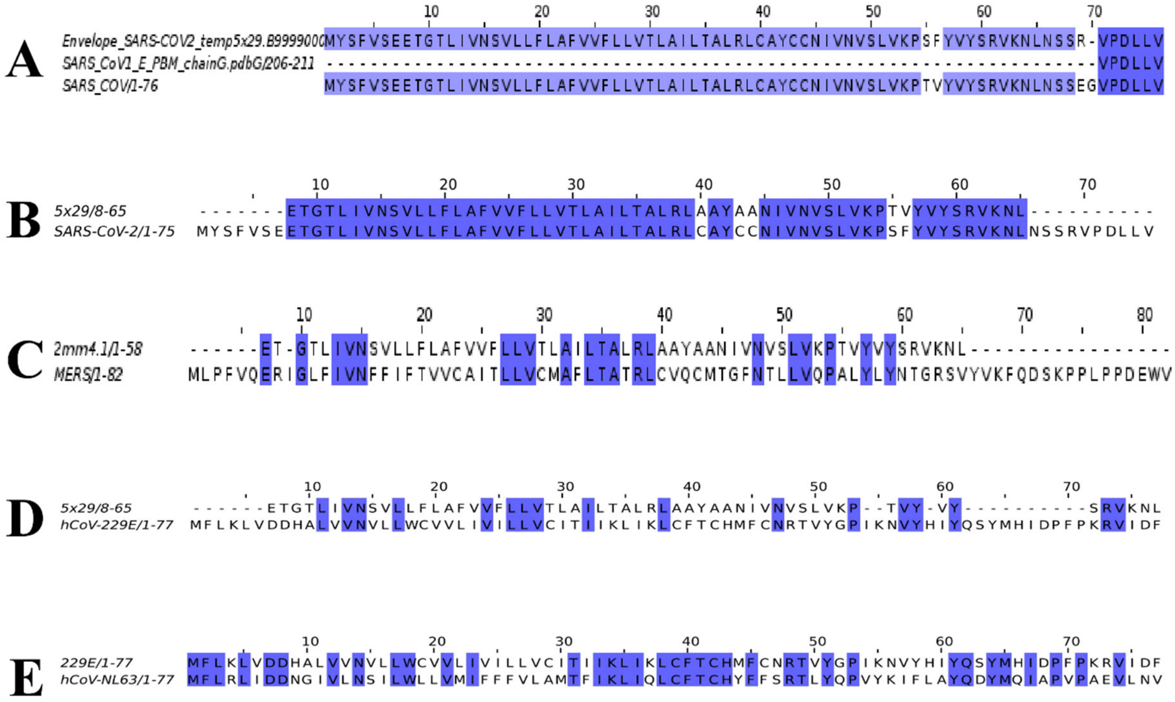

Figure S1. Pairwise sequence alignments between the more virulent SARS-CoV-1, -2 and MERS-CoV, and less virulent HCoV-229E and HCoV-NL63 human (h) coronavirus (CoV) envelope (E) proteins and the respective template used to generate each three-dimensional (3D) model. Sequence alignments were generated using Jalview (v2.11.1.3) and colored by sequence identity (blue). (**A**). Pairwise sequence alignment between the SARS-CoV-1 E protein (Accession number: P59637) and template 5X29. Sequences shared 91% identity with all residues conserved in the PDZ-binding motif (DLLV). (**B**). Pairwise sequence alignment between the SARS-CoV-2 E protein (Accession number: P0DTC4) and template 5X29. Sequences shared 91% identity with all residues conserved in the PDZ-binding motif (DLLV). (**C**). Pairwise sequence alignment between the MERS-CoV E protein (Accession number: K9N5R3) and template 2MM4. Sequences shared 35% identity with no conserved residues in the PDZ-binding motif (DEWV). (**D**). Pairwise sequence alignment between the HCoV-229E E protein (Accession number: P19741) and template 5X29. Sequences shared 29% identity and PDZ-binding motif residues VIDF were conserved. (**E**). Pairwise sequence alignment between the HCoV-NL63 E protein (Accession number: Q6Q1S0) and the HCoV-229E E protein homologous structure. Sequences shared 47% identity and PDZ-binding motif residues VLNV were conserved.

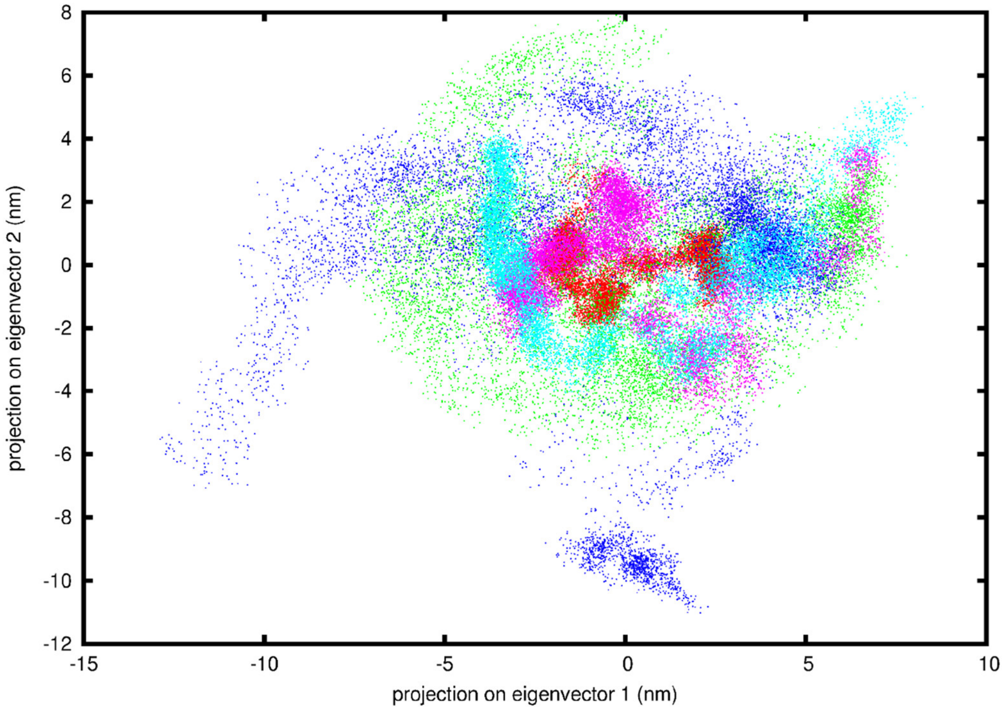

Figure S2. Principal component analysis (PCA) indicating the covariance matrix value of the human (h) coronavirus (CoV) envelope (E) proteins for SARS-CoV-1, -2, MERS-CoV, HCoV-229E, and HCoV-NL63 based on the projection of two eigenvectors. Red: SARS-CoV-2 E protein, Green: SARS-CoV-1 E protein, Blue: HCoV-229E E protein, Pink: HCoV-NL63 E protein, Cyan: MERS-CoV E protein.

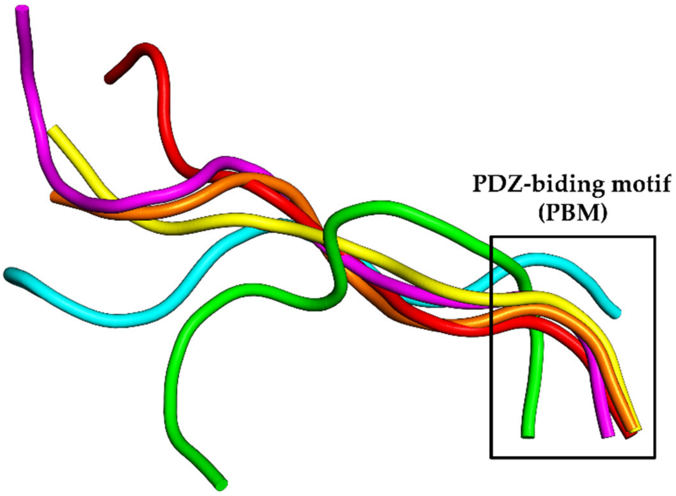

Figure S3. The C-terminals of the SARS-CoV-1, -2, and MERS-CoV envelope (E) protein models superimposed onto the C-terminals of the experimentally resolved SARS-CoV-1 and 2 E proteins. Orange: Experimentally resolved C-terminus of the SARS-CoV-1 E protein (PDB ID: 7NTJ), Yellow: Experimentally resolved C-terminus of the SARS-CoV-2 E protein (PDB ID: 7NTK), Pink: Experimentally resolved C-terminus of the SARS-CoV-2 E protein (PDB ID: 7M4R), Green: C-terminus of the SARS-CoV-1 E protein model, Red: C-terminus of the SARS-CoV-2 E protein model, Cyan: C-terminus of the MERS-CoV E protein model. The last four residues of the E protein C-terminal, corresponding to the PDZ-binding motif (PBM), is enclosed in the box.

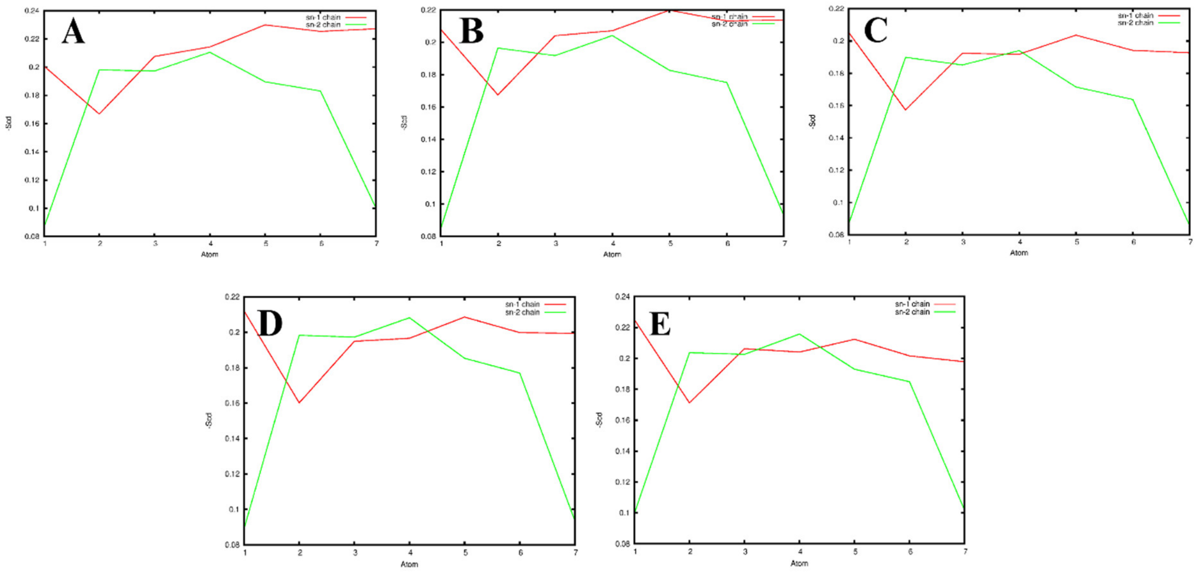

Figure S4. Deuterium order parameters of the sn-1 and sn-2 chains of the carbon acyl chains of the 1-palmitoyl-2-oleoylphosphatidylcholine (POPC) simulated lipid bilayer for the five human (h) coronavirus (CoV) envelope (E) protein systems. (**A**). SARS-CoV-2 E protein. (**B**). SARS-CoV-1 E protein. (**C**). MERS-CoV E protein. (**D**). HCoV-229E E protein. (**E**). HCoV-NL63 E protein.

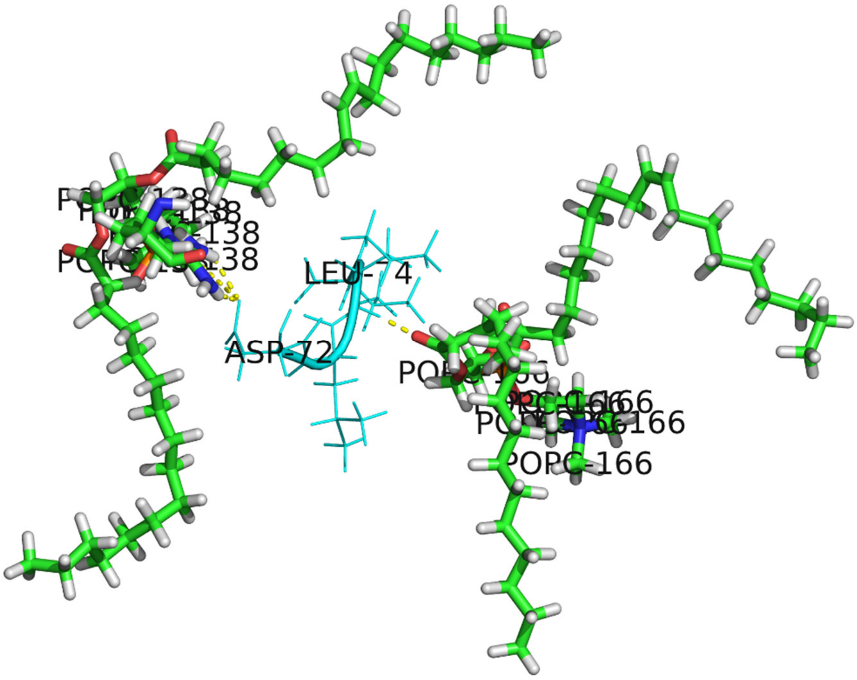

Figure S5. Interactions between the PDZ-binding motif (PBM) of the SARS-CoV-2 envelope (E) protein and the 1-palmitoyl-2-oleoylphosphatidylcholine (POPC) lipids in the simulated lipid bilayer. The interaction between the PBM of the SARS-CoV-2 E protein and the POPC lipids as visualized in PyMol (version 2.5.2). Cyan, licorice sticks and cartoon: PBM residues (**D**L**L**V) of the SARS-CoV-2 E protein. Green, sticks: POPC lipids (138 and 116). Yellow, dashed lines: polar contacts.

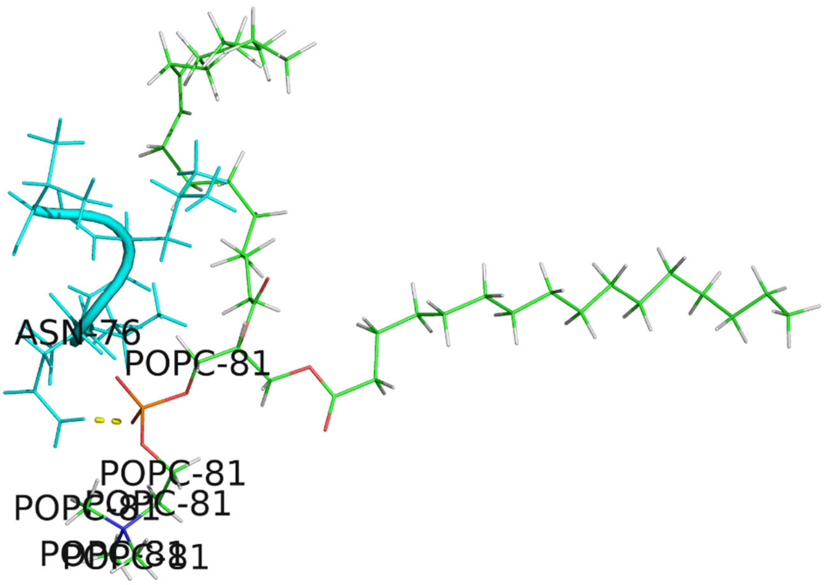

Figure S6. Interaction between the PDZ-binding motif (PBM) of the HCoV-NL63 envelope (E) protein and the 1-palmitoyl-2-oleoylphosphatidylcholine (POPC) lipids in the simulated lipid bilayer. The interaction between the PBM of the HCoV-NL63 E protein and the POPC lipids as visualized in PyMol (version 2.5.2). Cyan, licorice sticks and cartoon: PBM residues (VL**N**V) of the HCoV-NL63 E protein. Green, licorice sticks: POPC lipid (81). Yellow, dashed lines: polar contact.
